# Cancer-related microangiopathic hemolytic anemia (CR-MAHA) in a metastatic breast cancer patient with a germ-line ATM single nucleotide variant and an ESR1 fusion variant: insights from a case report on early diagnosis and improved outcomes

**DOI:** 10.3389/fonc.2026.1755142

**Published:** 2026-07-07

**Authors:** Fengting Yan, Aimee Wu, Yufei Wang, Tanya Wahl, Zirui Wang, Cesar Gutierrez, Josiah Wagner, Alexa Dowdell, Alexandra Bartlett, Christopher Carney, Daniel Landis, Brian Piening, Henry Kaplan

**Affiliations:** 1 Swedish Cancer Institute, Seattle, WA, United States; 2 University of Washington, Seattle,WA, United States; 3 The Ohio State University, Columbus, OH, United States; 4 Xi’an Jiaotong University Health Science Center, Xi’an, Shaanxi, China; 5 Providence Institute for Clinical Innovation, Portland, OR, United States; 6 Earle A Chiles Research Institute, Providence Cancer Institute, Portland, OR, United States; 7 Paul G. Allen Research Center, Seattle, WA, United States

**Keywords:** ATM, cancer-related microangiopathic hemolytic anemia, case report, ESR1, metastatic breast cancer

## Abstract

Cancer-related microangiopathic hemolytic anemia (CR-MAHA) is a rare and life-threatening paraneoplastic syndrome that carries a poor prognosis if not recognized early. While existing reports primarily focus on clinical outcomes, the underlying molecular changes remain poorly understood. We present a case in which the patient initially presented with back pain and fatigue. Further diagnostic workup confirmed the diagnosis of CR-MAHA, and she was promptly initiated on treatment, resulting in an excellent clinical anti-tumor response and resolution of the hemolytic anemia. Genomic testing revealed a germ-line ATM single nucleotide variant and an ESR1 fusion variant. This case raises important questions regarding potential genomic alterations that may predispose individuals to CR-MAHA and underscores the necessity for further investigation of tumor samples from these patients using both DNA and RNA sequencing.

## Introduction

Cancer-related microangiopathic hemolytic anemia (CR-MAHA) is a rare, life-threatening paraneoplastic syndrome characterized by hemolytic anemia resulting from microangiopathic changes, often occurring in the context of malignancies. It is most commonly associated with gastric cancer, followed by breast, prostate and lung cancer (1–7). This condition is marked by the destruction of red blood cells as they pass through microvascular networks altered by neoplastic processes, leading to the formation of fragmented red blood cells, or schistocytes, which are identified on peripheral blood smears.

The pathophysiology of CR-MAHA is not well understood and may involve a combination of factors, including the release of pro-inflammatory cytokines, direct tumor effects on vascular endothelium, and the activation of the coagulation cascade. These disturbances often result in the formation of microthrombi, contributing to a cascade of hemolytic processes, thrombocytopenia, and a variable presentation of clinical symptoms (1, 2). CR-MAHA can also be triggered by cancer therapies (e.g., mitomycin C, gemcitabine, targeted agents), which may cause direct endothelial toxicity or immune-mediated reactions (8, 9). Unlike classic thrombotic thrombocytopenic purpura (TTP), CR-MAHA typically does not involve severe ADAMTS13 deficiency and rarely responds to plasma exchange; treatment focuses on managing the underlying malignancy and discontinuing causative drugs (3–5).

Diagnosis of CR-MAHA requires a high level of clinical suspicion, especially in cancer patients presenting with signs of hemolytic anemia and thrombocytopenia. Rapid identification and management are crucial, as the condition can significantly impact treatment decisions and overall prognosis without effective treatment is extremely poor (3, 4). Treatment approaches may vary depending on the underlying malignancy and include addressing the cancer itself, managing hemolysis, and providing supportive care.

CR-MAHA represents a significant hematological complication in cancer patients, necessitating awareness and prompt intervention to improve outcomes in this vulnerable population. As our understanding of its mechanisms and associations deepens, further research and clinical vigilance will be essential in enhancing the management of this complex syndrome.

## Case report

A 39-year-old woman with a family history of breast and lung cancer presents for evaluation. Her mother was diagnosed with breast cancer at age 45, followed by metastatic lung cancer a few years later. Additionally, her maternal aunt had postmenopausal breast cancer. Genetic testing revealed an *ATM* c.7271T>G (p.V2424G) pathogenic mutation in the mother, and the patient underwent genetic testing in 2019, confirming the presence of the same *ATM* mutation. She established care with the high-risk clinic at University of Washington seven months prior to her presentation in September 2023. A multi-cancer panel (84 genes) was negative, except for the known *ATM* mutation. Notably, she had a negative mammogram while breastfeeding six months prior to her presentation.

She initially presented to her primary care physician with back pain and fatigue, which progressively worsened, leading her to visit a local emergency room in April 2024. Upon evaluation, she was found of having hemoglobin of 5.4 g/dL, a platelet counts of 38 K/uL, and elevated total bilirubin at 3.3 mg/dL. Additionally, her ALT and AST were elevated to over 200 U/L. A CT scan revealed extensive osteolytic lesions in the spine, although no discrete masses were identified. She received two units of packed red blood cell (PRBC) transfusion and was subsequently transferred to our center for further management.

Upon arrival, the patient only described back pain and fatigue. She denied other symptoms, including any central nervous system (CNS) symptoms. She was found to have a white blood cell count of 10.08 k/ul, hemoglobin of 8.0 g/dL, and a platelet count of 29 K/uL. Total bilirubin was elevated to 4.1 mg/dL, direct bilirubin of 1.8 mg/dL, indirect bilirubin 2.3 mg/dL, with AST at 246 U/L, ALT at 183 U/L and alkaline phosphatase at 182 U/L. Creatinine was normal at 0.78 mg/dL, with an estimated glomerular filtration rate (eGFR) more than 60. Haptoglobin was less than 10 mg/dL, lactate dehydrogenase (LDH) was 2,881 U/L, and uric acid was 8.3 mg/dL. The reticulocyte count was elevated at 7.1%, and the direct antiglobulin test (DAT) was negative.

**Figure 1:**
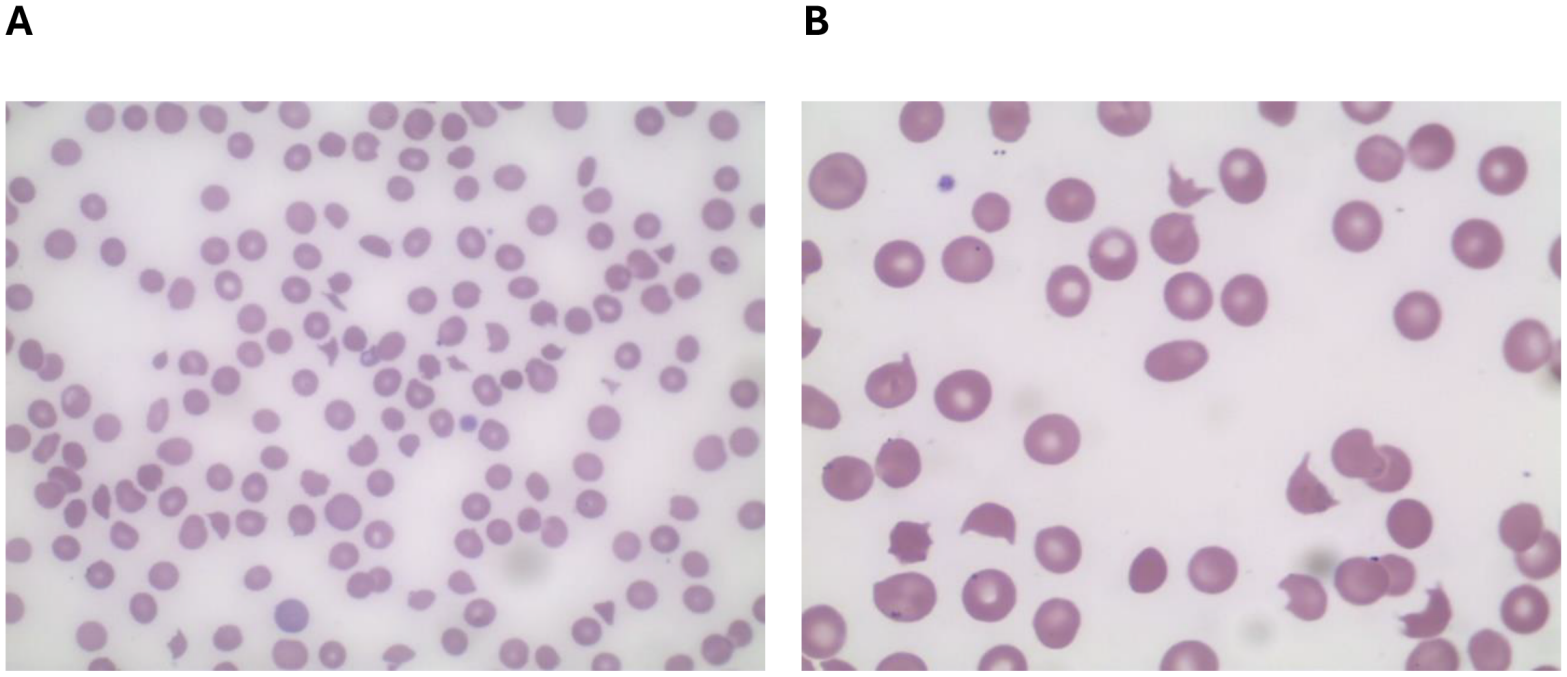
Peripheral blood smear showing fragmentation of red blood cells. **(A)** 60x and **(B)** 100x.

The chronic hepatitis panel was negative, except for a reactive hepatitis B surface antibody. The DIC panel showed occasional schistocytes, an elevated D-dimer of 6.95 ug/mL, and normal prothrombin time, partial thromboplastin time (PTT) and fibrinogen levels. The peripheral smear indicated thrombocytopenia, a left shift of polymorphonuclear leukocytes, numerous red cell fragments, and the presence of nucleated red blood cells, with no blasts observed on scanning (**Figure 1**). ADAMTS13 was greater than 100 VWF cleaving protease actual/normal.

**Figure 2:**
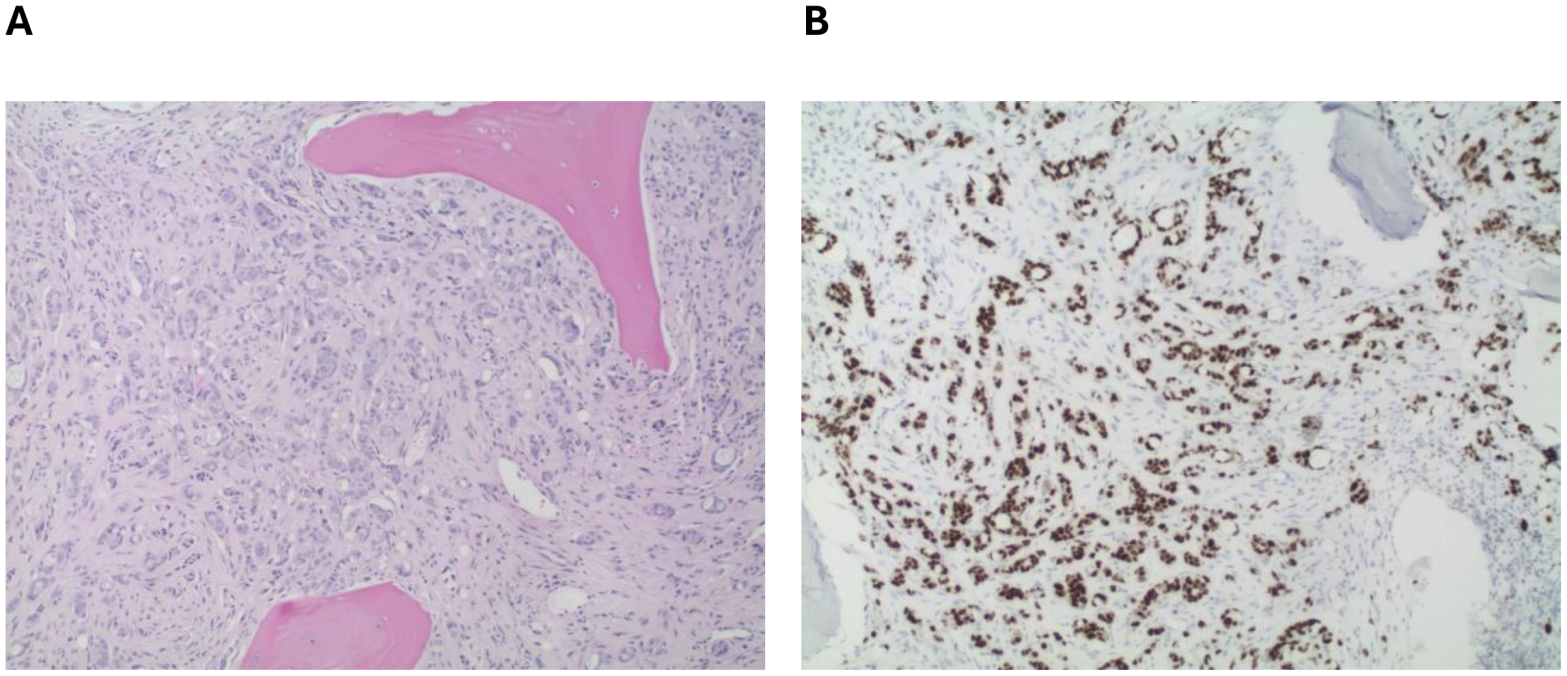
Core bone marrow biopsy showing **(A)** Tumor Cell infiltration of marrow by H&E and **(B)** Positive GATA3 stain. Negative TTF-1 stain not shown.

**Figure 3:**
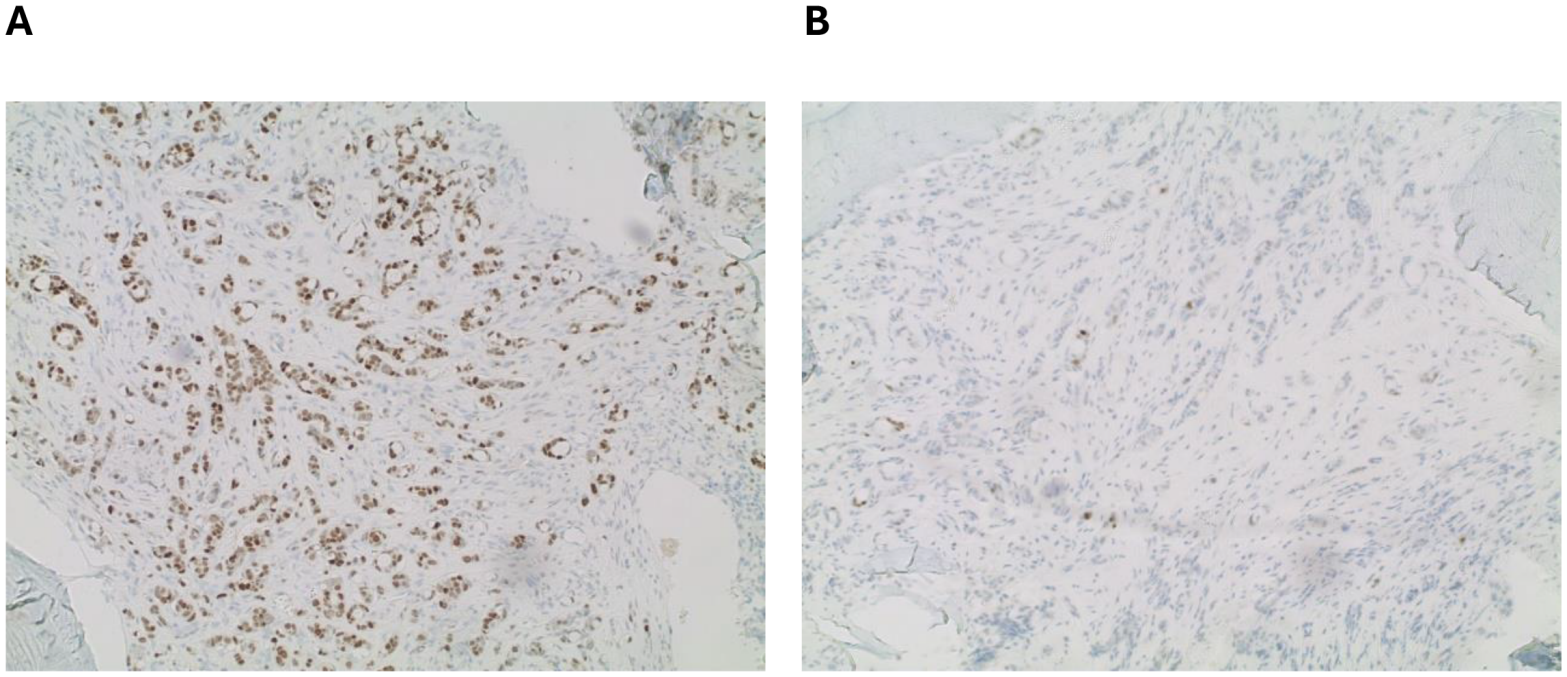
**(A)** Estrogen receptor and **(B)** progesterone receptor stains of tumor cells in core bone marrow biopsy. 1+ HER2 IHC stain not shown.

Bone marrow biopsy was performed on the day of admission due to clinical suspicion of CR-MAHA. Bone marrow aspirate smears showed bloody smears with a single cluster of metastatic carcinoma, iron stain was normal. Bone marrow core biopsy revealed metastatic adenocarcinoma consistent with a breast primary, GATA3 was positive (**Figure 2**) and TTF-1 was negative. Estrogen receptor (ER) expression was moderate to strong, at 99%, while progesterone receptor (PR) staining was weak to moderate strong, at 5% (**Figure 3**). Her2 was negative, showing 1+ by immunohistochemistry (IHC).

A PET/CT revealed a left breast mass and left axillary lymphadenopathy. Additionally, diffuse heterogeneous uptake was noted throughout the axial skeleton and proximal appendicular skeleton, involving most of the visualized bones.

She was initiated on a weekly regimen of doxorubicin at 20 mg/m2 and cyclophosphamide at 200 mg/m2 within 48 hours of her presentation to our facility. Additionally, she received zoledronic acid on day 4. The chemotherapy resulted in a positive response, with improvements noted in her hemoglobin, platelet count, and bilirubin levels. She last received PRBC transfusion on day 13, has not required any transfusion since then, and was discharged home on that day.

She received two additional doses of weekly doxorubicin at 20 mg/m2 and cyclophosphamide at 200 mg/m2 as outpatient, followed by one additional chemotherapy infusion of doxorubicin at 60 mg/m2 and cyclophosphamide at 600 mg/m2. This treatment was complicated by mucositis and febrile neutropenia, during which she was found to have a rhinovirus infection, from which she recovered without difficulty.

**Figure 4:**
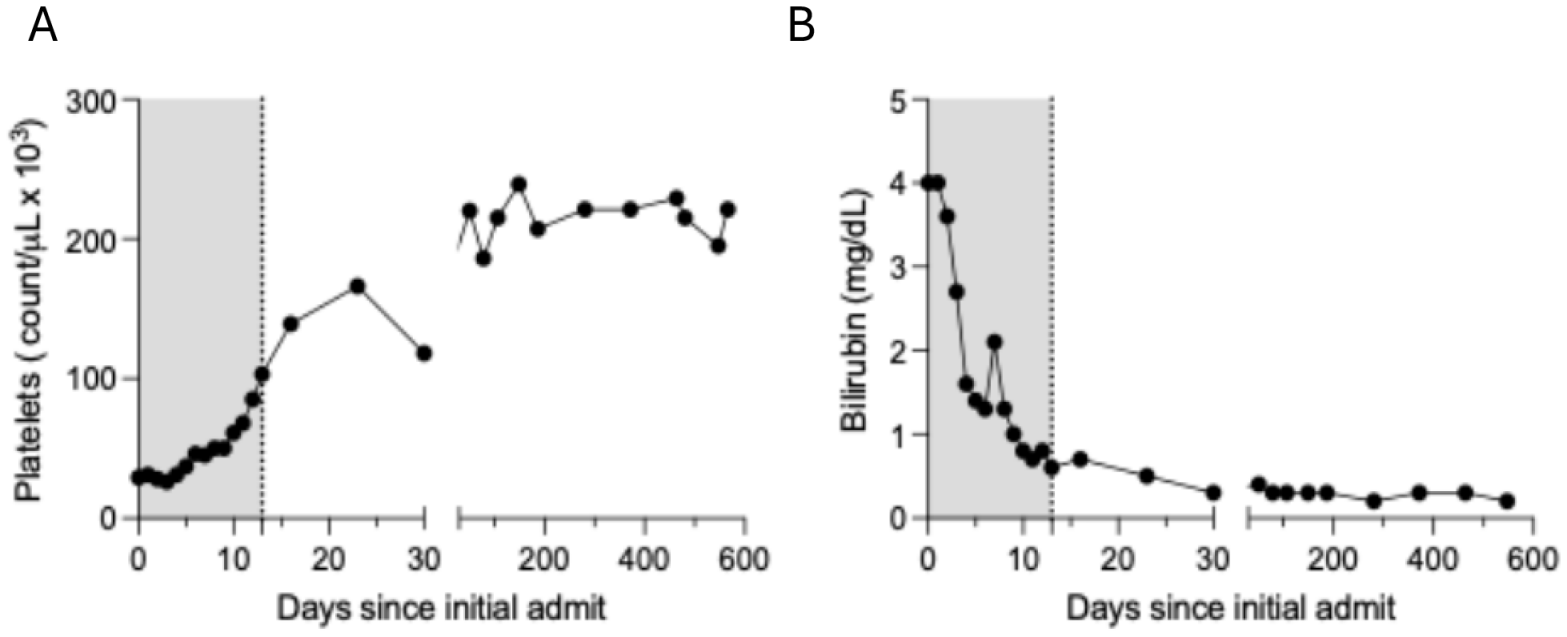
Time course of improvement in platelet count and bilirubin with chemotherapy. Improvement of platelet count **(A)** and total bilirubin **(B)** after chemotherapy was initiated as inpatient. Grey shading indicates inpatient time and dotted line indicate hospital discharge.

**Figure 5:**
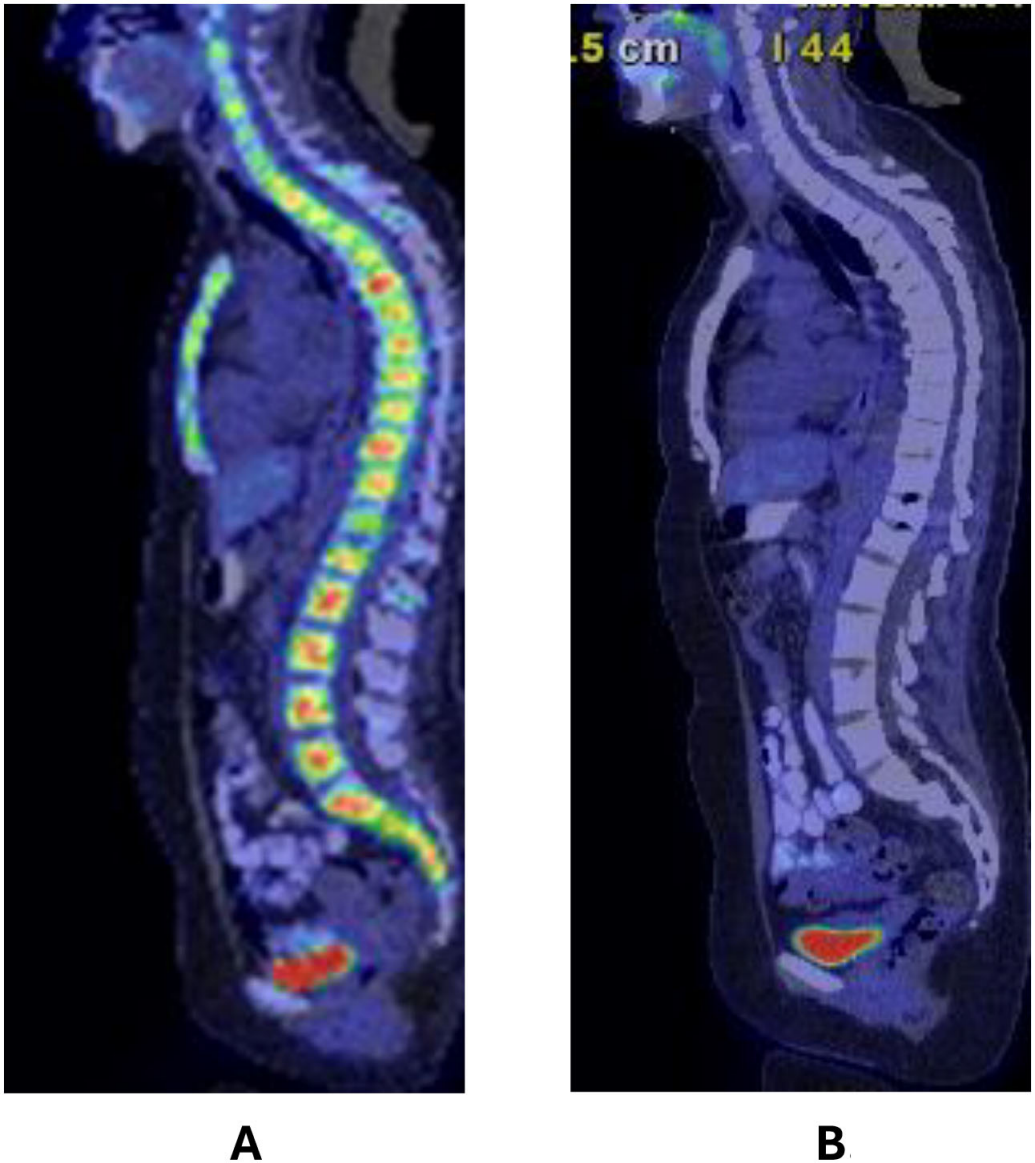
Improvement in tumor deposits in bone with therapy by PET/CT scan. PET/CT before and after treatment. **(A)** Before treatment **(B)** 6 months after treatment.

She began ovarian function suppression (OFS) with a leuprolide acetate injection seven weeks after the start of anticancer therapy and changed antitumor therapy to anastrozole and abemaciclib eight weeks after diagnosis. The rapid improvement in platelet count and bilirubin are depicted in **Figure 4** and the improvement in PET/CT scan is noted in **Figure 5**. She continued OFS with anastrozole and abemaciclib but was found to have interval osseous progression involving T9, right iliac wing, right acetabulum and trochanteric region of the left femur on PET/CT in July 2025, 13 months after starting hormone therapy. As a result, she received stereotactic body radiation therapy (SBRT) to the T9 lesion, and anastrozole was switched to fulvestrant in September 2025. She has remained stable since then.

The patient provided consent to participate in a serial genomic study utilizing solid tumor and liquid biopsy comprehensive genomic profiling as well as DNA methylation studies. The study utilized the ProvSeq 523 clinical lab developed test based upon the TruSight Oncology 500 High Throughput assay and detects DNA variants in 523 genes and RNA fusions in 55 genes from formalin-fixed, paraffin-embedded (FFPE) samples (10, 11). The bone marrow tissue sample tested using ProvSeq 523 indicated the presence of an *ATM* exon 49 mutation (c.7271T>G:p.V2424G) in the DNA results and an *ESR1-CCDC170* fusion variant in the RNA results. The *ESR1-CCDC170* fusion is predicted to consist of a fusion between *ESR1* exon 2 and *CCDC170* exon 6, resulting in N-terminally truncated CCDC170 protein (PMID: 36686845, PMID: 25099679). The tumor mutational burden was classified as low, with 2 mutations per megabase, and the tumor was determined to be microsatellite stable. Additionally, the ProvSeq 523 analysis returned negative results for clinically significant mutations in *AKT1*, *BRCA1*, *BRCA2*, *ERBB2* (HER2), *PIK3CA*, *NTRK1-3*, and *RET*. Multiple blood samples over the next year were tested using a liquid biopsy assay (TSO500 ctDNA). However the assay does not test for *ESR1* fusion variants. The patient underwent a Guardant 360 CDX test in January 2025, which returned positive only for the *ATM* V2424G mutation, with a circulating free DNA (cfDNA) percentage of 48.7%. The Guardant assay also does not test for *ESR1* fusion variants. In-house germline testing of the patient using whole exome sequencing from whole blood DNA extract suggests the *ATM* V2424G variant is germline origin. It is classified as consensus Pathogenic for ATM-related cancer predisposition by a ClinGen expert panel (https://www.ncbi.nlm.nih.gov/clinvar/variation/3023/0).

## Discussion

Here, we present an unusual case of cancer-related microangiopathic hemolytic anemia (CR-MAHA), which is often not quickly recognized, with untreated patients typically having a life expectancy of only days to weeks (12, 13). In this case, however, the condition was identified early, and treatment was initiated promptly. With induction chemotherapy followed by CDK 4/6 inhibitor/hormone treatment she has thus far done extremely well for 26 months.

Early recognition and prompt initiation of treatment for CR-MAHA requires a high index of suspicion in cancer patients presenting with evidence of anemia. Clinical recognition begins with evaluation of anemia in cancer patients when hemoglobin is ≤11 g/dL or there is a ≥2 g/dL drop from baseline. For patients without a cancer diagnosis, CR-MAHA should still be considered in the differential diagnosis. Diagnostic workup should include a complete blood count (CBC) with platelet count and indices, reticulocyte count, lactate dehydrogenase (LDH), haptoglobin, renal and liver function testing (including indirect bilirubin), and a direct Coombs test. The peripheral blood smear is critical for identifying schistocytes, which are diagnostic of microangiopathic hemolytic anemia. Early bone marrow biopsy is recommended if malignancy is suspected and the diagnosis remains unclear. Exclusion of other thrombotic microangiopathies, such as thrombotic thrombocytopenic purpura (TTP), hemolytic uremic syndrome (HUS), disseminated intravascular coagulation (DIC) and drug-induced thrombotic microangiopathy (TMA) is necessary, using ADAMTS13 activity, coagulation studies, and clinical context. The lack of renal and central nervous system dysfunction has been noted in CR-MAHA, which, while not definitive, may help suggest CR-MAHA as opposed to TTP or HUS in a cancer patient (5).

CR-MAHA is distinguished from other TMAs by its poor response to plasma exchange and its association with advanced cancer. With regard to breast cancer, it has been reported in both ductal and lobular disease and in both hormone receptor positive and triple negative disease; It has always been reported in association with stage IV disease (6, 14). Bone marrow involvement is very common, though not universal (1, 15). In a study of 54 breast cancer patients with CR-MAHA, the median interval from the diagnosis of metastatic disease to the onset of CR-MAHA was 16.7 months. The median overall survival from onset of MAHA was 28 days but early recognition and prompt initiation of therapy can sometimes improve outcomes. Approximately 10% of patients in this study survived for more than one year (6).

This case is also interesting in that genomic sequencing of this patient’s bone marrow detected both an ATM single nucleotide variant and an *ESR1-CCDC170* fusion variant present at the time of diagnosis. The ATM variant is known to cause ataxia telangiectasia in homozygous carriers and to increase the risk of breast cancer (16). The *ESR1-CCDC170* fusion variant, though rare, is the most frequently detected fusion variant in luminal B breast cancers (17, 18). To our knowledge, neither variant has been associated with CR-MAHA. Of note, while the *ATM* variant was detected across germline, solid tumor and liquid biopsy assays, the *ESR1-CCDC170* fusion was only detected via RNA sequencing in the solid tumor assay, whereas both popular liquid biopsy assays (Guardant 360 and TSO500) were not designed to detect this variant. It is likely that additional capture probes around the fusion breakpoints would need to be added to either assay to enable this detection in the future. Alternately, techniques for RNA sequencing in circulating tumor cells (19) and cfRNA sequencing in plasma (20) from cancer patients have been developed and could be adapted for these purposes.

There is little clinical data regarding genomic alterations that might be associated with CR-MAHA. It would be particularly useful to assess whether this variant, or any others, are associated with the occurrence of CR-MAHA. This patient’s particular variant is associated with a more aggressive course of breast cancer as well as with hormone therapy resistance (21, 22). However, there is only a modest amount of clinical data in this setting regarding the use of the variety of hormonal agents currently available. One tantalizing observation by Li et al (17) is that ESR1-CCDC170 binds to HER2/HER3/SRC and activates SRC/PI3K/AKT signaling in the two breast cancer cell lines studied. These cells were resistant to tamoxifen and fulvestrant but were highly sensitive to treatment regimens combining tamoxifen or fulvestrant with the HER2 inhibitor lapatinib and/or the SRC inhibitor dasatinib (17). To our knowledge this has not been attempted in patients at this time. It will be important to develop experience with both chemotherapy, modern hormone therapies and novel combinations for this variant but our ability to do so is limited by its rarity.

Building on the established preference for CDK4/6 inhibitors plus endocrine therapy, recent meta-analyses and real-world data confirm that CDK4/6 inhibitors (palbociclib, ribociclib, abemaciclib) combined with endocrine therapy significantly improve progression-free and overall survival compared to endocrine monotherapy, regardless of menopausal status, treatment line, or extent of metastatic disease in hormone receptor positive patients (23–25). This combination is effective even in patients with visceral involvement, unless there is a true visceral crisis requiring rapid cytoreduction. Chemotherapy is generally reserved for patients with rapidly progressive, symptomatic visceral crises or those who have exhausted endocrine and targeted options (26). Visceral crisis is defined by severe organ dysfunction and imminent threat to life, not merely the presence of visceral metastases. In these scenarios, cytotoxic chemotherapy is preferred for its faster onset of action. In this case, CR-MAHA was a crisis requiring urgent treatment and inpatient chemotherapy.

## Conclusion

CR-MAHA is a rapidly progressive complication of metastatic malignancy seen most commonly in gastric or breast cancer. Early diagnosis and prompt treatment are crucial for improving the survival of these patients. The current case raises the question of whether there are genomic alterations that might predispose to this condition and the need to study these patients’ tumors with both DNA and RNA sequencing.

## Data Availability

The original contributions presented in the study are included in the article/Supplementary Material. Further inquiries can be directed to the corresponding author/s.
